# Disease of the Pancreas

**Published:** 1890-10-04

**Authors:** 


### THE PRACTITIONER'S MIRROR AND RETROSPECT.

[*The Editor will be glad to receive offers of co-operation and contributions from members of the profession. There is no doubt that much valuable material full of interest and instruction is at present lost to science. This is largely so in the case of* (1) *the younger and less-known members of the hospital staff* (2) *those connected with cottage hospitals, and* (3) *the great body of medical practitioners not attached to any institution. The co-operation of all is cordially invited to enable the Editor to make* THE HOSPITAL *of the utmost possible use and value to the profession. Specialists willing to review books on their special subjects are invited to communicate this fact at once. All letters should be addressed to* THE EDITOR, THE LODGE, PORCHESTER SQUARE, LONDON, W.]

### MEDICINE.

### DISEASE OF THE PANCREAS.

Dr. Frederick Roberts observes that owing to the comparative rarity of diseases of the pancreas, their frequent association with other lesions, the position and relation of the organ, there is no part of the body, the diseases of which it is more difficult to recognize. Perhaps this in the reason why we hear so little about them. Persons often talk of their stomach, and liver, and bowel complaints, but we do not recollect even a hypochondriac ever speaking of his pancreas, as the organ affected! And we never heard of anyone taking medicine to act on his pancreas, as we are in the habit of hearing of people taking liver pills, &c. Even the fraternity of nostrum advertisers have not yet discovered the pancreas pill! Nevertheless the pancreas plays an important part in the interior economy, and especially in the process of digestion. From the writings of older authors it is manifest that more importance was formerly attached to this organ than in modern days. Thus Schenkins stated "the pancreas and intestines are the seat of numerous and wonderful diseases." Hernelius considered it the seat of diarrhœa, dysentery, cachexia, atrophy, langour, and slow fever. Riolanus supposed hypochondriasis and other chronic diseases were excited by its obscure maladies. Although our knowledge of human physiology and of chemistry is now much more accurate than in bygone times, it has not resulted in perfect knowledge of the functions of the pancreas. We know that the gland secretes a fluid which enters the alimentary canal by opening close to the bile duct. The pancreatic fluid is said to contain three hydrolytic ferments: a petone-forming ferment, *trypsin*; a fat-splitting ferment, *steapsin*; and a diastatic ferment, *amylopsin*. It may be presumed that this is correct. But the collection of pancreatic juice is attended with much difficulty. The duct is nearly always found empty after death. And even were this not the case, the juice being always in comparatively small amount there is no doubt that after death it becomes quickly deteriorated. If taken from animals it can only be obtained by operation. Bernard, for instance, confessed that he obtained pancreatic juice from irritated and inflamed glands because they secreted it faster. Hence the descriptions given by different authors of pancreatic juice have varied to a considerable extent. Now, we venture to doubt the correctness of the opinion that diseases of the pancreas are rare, and we believe that many cases are returned as fatal from other causes, when a *post-mortem* examination would disclose the pancreas as the organ mainly or only affected. As with other organs, so with the pancreas, diseases may be classed under the three heads of functional, structural, and malignant. Under these three heads there are at least twenty-five diseases of the pancreas which have been noticed. Now the gland being subject to a formidable catalogue of affections, we can only suppose that the difficulties of diagnosis are so great as to mislead many practitioners. Of pancreatic disease there are few signs proper, and the test-tube, the microscope, and the stethoscope are for the most part useless. A certain amount of inductive reasoning is requisite before the pancreas can be fixed upon as the organ in fault. There is, however, one malady of the pancreas the symptoms of which are tolerably well established. Dr. Fitz, of Boston, collected 70 cases of acute pancreatitis. He distinguishes three forms—purulent, hæmorrhagic, and gangrenous. It almost always attacks strong corpulent males. The onset is generally sudden, with headache, giddiness, vomiting, distension of the abdomen, stabbing pains, and prostration. It is usually fatal, and often so in a few hours. The diagnosis lies between it and irritant poisoning, perforative peritonitis, and acute intestinal obstruction. Lanquenhaus showed, in the latter part of last year, to the Medical Society of Berlin, a typical case of pancreatitis, or, as he terms it, "necrosis of the pancreas." The symptoms were sudden giddiness, vomiting, swollen abdomen. and palpitation, followed by collapse. This patient, however, lived six weeks, and some spots presenting on the abdomen caused the case to be diagnosed as one of enteric fever. On *post-mortem* examination "a cavity was found, in which the pancreas lay quite separate, like a sequestrum," the texture of the gland being very soft and brittle.

It is probable, however, that the functional diseases of the pancreas are the most frequent. There may be increased secretion, diminished secretion, or secretion of a depraved juice. It is generally admitted that whatever excites the salivary glands will act in a similar manner on the pancreas. Hence there is reason to believe that the habitual use of tobacco may cause hyper-secretion by the pancreas. Similarly the habitual use of mercurial pills or of iodide of potassium may do the same. Annesley, the author on Indian diseases, long since stated that the habitual use of heating and irritating articles of diet will perform their part in the production of hyper-secretion by the pancreas. This has been supposed to give rise to a form of pyrosis. But that this does not always, or even commonly, result is very probable, from the following reasons. If we turn to works on anatomy we find Ellis stating that the pancreatic duct opens either conjointly with the common bile duct or by a separate aperture. But in many instances the pancreatic duct opens into the ductus communis choledochus, or even into the gall bladder. When this occurs the pancreatic secretion must become immediately incorporated with the bile. Pyrosis, therefore, cannot occur, although so-called bilious vomiting may result. Chronic diarrhœa has also been attributed to excess of pancreatic secretion, which formerly received the name of *fluxus pancreaticus*. But pancreatic juice has been administered and found not to act as a laxative. Still, diarrhœa may occur from anything which irritates the bowels, whether it be an excess or diminution of a natural secretion. There are, however, no distinctive symptoms of diarrhœa from excess of pancreatic secretion. A *diminished* secretion of pancreatic fluid has been connected with the presence of free fat or oily matter in the stools, which has not been digested owing to the want of pancreatic fluid. This was first noticed as a symptom of impaired function of the pancreas by C. Bernard, who produced it by experiments on animals. It has also been noticed clinically, especially by Kuntzmann, Unckel, Fitz, and others. The fatty stools persist even when fat has been withdrawn from the food, and sometimes there is associated with it fatty vomiting. There may, however, be severe pancreatic disease without fatty stools, and it is more likely to occur when the entrance of bile into the intestine is also interfered with. And it should not be forgotten that fat has been passed in large quantities without any pancreatic affection, not only *per rectum* but also *per urethram*. It is certain that this fatty material may occur in the evacuations with or without pancreatic disease. Of depraved or altered pancreatic juice nothing is known.

Glycosuria has been produced experimentally in animals by destruction or injury of the pancreas, and according to Lancereaux and Lapierre, it may be evidence of pancreatic disease. Mons. Lancereaux asserts there are three forms of diabetes, viz., the nervous, that occurring in the obese, and the emaciative form, the latter being due to chronic pancreatitis or pancreatic lithiasis. On the other hand Schnée, in his recent work on diabetes, does not connect it with the pancreas. It is, however, certain that in some instances diabetes has preceded pancreatic disease, and that in other instances it has been a sequence. But in such cases the diabetic condition probably depends not on the pancreas, but on the implication of the solar and cæliac plexuses, and semi-lunar ganglia. Lancereaux, however, says that diabetes with emaciation consequent on diseases of the pancreas, comes on suddenly, the amount of sugar in the urine is large, and the duration of the disease not long, the patients usually dying of tuberculosis. Boils and carbuncles, he says, do not form as in the diabetes of the fat.

Chronic diseases of the pancreas are numerous. There are chronic inflammation, hypertrophy, and atrophy, abscess, tubercular deposit, calculi, serous cysts, hydatids, fatty deposits, superficial lymph deposit, hæmorrhage, induration, softening, gangrene, and the different forms of cancerous disease. It may be said that chronic pancreatic disease is marked by abdominal pain, swelling, and sometimes pulsation, vomiting, thirst, emaciation, vacarious secretion of the salivary glands, often jaundice, and reflected affections of the nervous system, as insomnia, for instance. Yet none of the symptoms are decidedly distinctive.

Carcinomatous diseases of the pancreas occur usually in the head of the gland in that extremity which lies nearest the bowel. This may cause jaundice by obstructing the bile duct. And it may occasion great enlargement of the liver. It may also produce distension of the stomach by compressing the duodenum and preventing the free passage of aliment through the gut. But the same may result from similar disease in adjacent organs, and, as a matter of fact, carcinomatous disease of the pancreas is often found in connection with carcinomatous disease elsewhere. Graham Brown, in his last edition of "Medical Diagnosis," says that tumours of the pancreas, which are almost invariably carcinomatous, are difficult of diagnosis owing to the way in which the gland is covered by the coils of the intestines, and to some extent by the lower edge of the liver. But hardness in such cases can usually be felt to the right of the middle line above the level of the umbilicus. It is deeply seated, not freely moveable, and unaffected by respiratory movements. If it is carcinoma, it is seldom limited to the pancreas, but attacks the lymphatic glands and other parts.

Dr. Watson said, in those admirable lectures, which were in former years the text-book for medical students, that it may seem a slight to the pancreas to pass it over without noticing the diseases to which it is subject; and he seemed to think it ought to behave better, for he complained "the diseases do not signify their existence by any plain or intelligible signs." As to remedies for pancreatic diseases or disorders, "I do not know of any." And another author remarked, that "the remedies for a diseased pancreas are as imperfect as the symptoms which mark its derangement." When there is acute disease the most obvious indications are rest, liquid food, ice, laxatives and stimulants if required. It has also been recommended to apply leeches, blisters, or fomentations, near the salivary glands, and so endeavour to excite a metastasis of inflammatory action to those sympathising organs, but we are not aware that this has proved beneficial. In chronic pancreatic disease, while using those remedies, indicated by existing symptoms, it may be well not to be unmindful of the plan pursued by the older physicians. When they met with cases in which stomach pain, or pain in some neighbouring viscus, was chiefly complained of, and when good evidence of the nature of the disease was not forthcoming, they regarded maintaining the *prima via* clear, as the principal object.

As regards operative procedure, Mr. Meredith says in his recent address "on the present position of abdominal surgery, premeditated radical operations on the pancreas have been rarely attempted in consequence of its deep-seated position, and its close and varied connections with other important abdominal structures. Its removal by extirpation has till now proved all but invariably fatal." But Mr. Meredith adds, on the other hand, treatment of simple or hydatid cysts and of suppurations in connection with the organ by free incision, followed by suture of the sac to the abdominal wall, and drainage of its cavity, appears to have yielded excellent results. In the *British Medical Journal* for May 3rd, is a communication from Dr. Christian Simpson, remarking on an observation of Mr. Treeves, that no operation on the pancreas had been performed in this country, for the disease had not been diagnosed. Dr. Simpson refers to a case reported by himself in 1889 of a pancreatic cyst which was diagnosed as such in 1885, and subsequently treated by incision and drainage resulting in cure.

